# Research on an Identification Method for Wheelset Coaxial Wheel Diameter Difference Based on Trackside Wheelset Lateral Movement Detection

**DOI:** 10.3390/s23135803

**Published:** 2023-06-21

**Authors:** Xinyu Peng, Jing Zeng, Qunsheng Wang, Haiyan Zhu

**Affiliations:** 1 Traction Power State Key Laboratory, Southwest Jiaotong University, Chengdu 610031, China; peng@my.swjtu.edu.cn (X.P.); qs_wang@swjtu.edu.cn (Q.W.); 2 School of Mechatronics and Vehicle Enginneering, East China Jiaotong University, Nanchang 330013, China; 2562@ecjtu.edu.cn

**Keywords:** wheelset coaxial wheel diameter difference, online monitoring, railway freight cars, dynamics, lateral movement

## Abstract

The wheelset coaxial wheel diameter difference is one of the most common wheel faults of railway vehicles. The existence of the wheelset coaxial wheel diameter difference may lead to the off-load operation of vehicles, resulting in abnormal wheel tread wear, leading to the deterioration of the wheel–rail contact relationship, resulting in the deterioration of the vehicle’s operating stability and comfort, and even leading to an increase in the derailment coefficient, affecting the running safety. In order to monitor the freight car wheelset coaxial wheel diameter difference online, a vehicle–track coupling dynamics model based on a trackside detection method was established, and the response of rail lateral displacement under the condition of the wheelset coaxial wheel diameter difference was analyzed. The results show that the existence of the wheelset coaxial wheel diameter difference can lead to a deviation in the vehicle’s run, with an increase in the wheelset coaxial wheel diameter difference and an increase in the lateral offset of wheelset increases. The impact of vehicle unbalance loading on the lateral movement of the wheelset is much smaller than that of the wheelset coaxial wheel diameter difference. The existence of the wheelset coaxial wheel diameter difference can be better reflected by detecting the wheelset’s lateral displacement. On straight line, the variation of lateral displacement has no infection of vehicle speed, but shows a quadratic growth trend with the wheelset coaxial wheel diameter difference. Based on this, the mapping relationship between the wheelset coaxial wheel diameter difference and wheelset lateral displacement can be obtained. Through a mapping relationship, the size of the wheelset coaxial wheel diameter difference can be reversed precisely through the detection of a trackside lateral movement monitoring system. The reliability of the identification method was verified with a specific test on the trackside monitoring system.

## 1. Introduction

**Figure 1:**
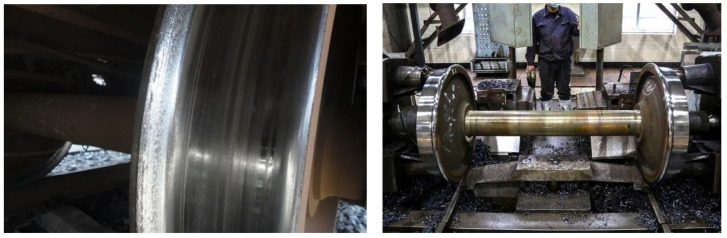
Severe wheel tread wear (**left**) and rotary repair of wheelset (**right**).

Heavy haul railways have always been an important part of China’s railway development. With the continuous increase in the axle loads of railway freight transport vehicles, the loads of vehicles on the track also increase gradually. During the running of the wheelset, the uneven wear of the left and right wheels results in a wheelset coaxial wheel diameter difference, which refers to a deviation in the rolling circle diameter of the left and right wheels of the wheelset. The appearance of the wheelset coaxial wheel diameter difference not only leads to unbalanced loads of vehicles and aggravates the partial wear of wheels but also causes a serious impact on rails, track beds, etc. [1], and even endangers traffic safety. Because freight cars are generally large, when a wheelset coaxial wheel diameter difference occurs, the left and right-side wheel rail force difference of the wheel is much larger than that of the passenger train, and the damage to the track is greater. Therefore, in the maintenance of railway freight cars, the regulation of the wheelset coaxial wheel diameter difference is very strict. Due to wheel wear (Figure 1 left), wheel rotation errors (Figure 1 right) and other unavoidable reasons, vehicles inevitably produce a wheelset coaxial wheel diameter difference. Most of the traditional wheelset coaxial wheel diameter difference detection means mainly rely on the use of wheel diameter ruler. Generally, it take place in the vehicle depot maintenance, and the bogie and the body need to be separated. The traditional detection methods not only consumes a lot of manpower and material resources, and it is difficult to achieve the effect of monitoring the wheelset coaxial wheel diameter difference.

The efficiency of monitoring the wheelset coaxial wheel diameter difference can be greatly improved by using intelligent devices, but the development of intelligent devices relies on the theoretical basis of the dynamic response of the wheelset coaxial wheel diameter difference. In recent years, many scholars have conducted specific research on the wheelset coaxial wheel diameter difference. Li et al. [2] studied the influence of grooved treads and the wheelset coaxial wheel diameter difference on vehicle dynamics, and they analyzed the formation of grooved treads with a method for wear prediction. Sawley and Wu [3] conducted a numerical analysis of the wheel–rail relationship of grooved treads after investigating the wear of grooved treads in North America, and the results showed that grooved treads are a result of the deterioration of the wheel–rail relationship, which leads to an increase in the lateral force of the axle and the deterioration of the vehicle’s dynamic performance. In addition, grooved treads are caused by the partial wear caused by differences in the wheel diameter. Sawley [4] also studied the influence of notched treads on vehicle stability. Lyu et al. [5,6] studied the influence of the initial wheelset coaxial wheel diameter difference on wheel surface fatigue damage through field investigation and numerical simulation, and the results showed that the wheelset coaxial wheel diameter difference causes cracks in side wheels with smaller diameters, causing local wear and aggravating crack initiation. Sui et al. [7] studied the influence of the wheelset coaxial wheel diameter difference by applying the Non-Hertz contact theory on the basis of on-the-spot investigation on the wear state of the wheelset tread. The results showed that the initial wheelset coaxial wheel diameter difference causes the shape of the contact zone and the distribution of stress in the contact zone, leading to the asymmetric wear of the wheelset and changes in the wear rate of the tread. Chen et al. [8] studied the influence of the wheelset coaxial wheel diameter difference with different amplitudes and distributions on the wheel–rail contact relationship and the vehicle’s dynamic performance in a turnout area, and the results showed that the wheelset coaxial wheel diameter difference aggravates the natural structure’s lack of smoothness in the turnout area and greatly affects the normal force at the sharp rail. It was suggested that the wheelset coaxial wheel diameter difference should be controlled within 2 mm.

Chi et al. [9] analyzed the force of the bogie with a wheelset coaxial wheel diameter difference and theorized that the existence of the wheelset coaxial wheel diameter difference changes the alignment balance position of the wheelset, thus changing the wheel–rail contact relationship and affecting the stability of the vehicle system. He et al. [10] simulated and analyzed the lateral wheel–rail force of the bogie wheelset of a C0-C0 locomotive in 2011 and simulated the change in the wheel–rail force when the wheel diameter difference occurred in the wheelset and upon the locomotive braking. This study verified the real operation situation in which a wheel diameter difference occurs in a locomotive during actual operation. However, the mapping relationship between the wheelset coaxial wheel diameter difference and wheel–rail force has not been discussed in detail. Zhu [11] analyzed the force effect of the wheelset coaxial wheel diameter difference on the wheel wear of a rail vehicle in 2017, aiming at the influence of the wheelset coaxial wheel diameter difference on the bias wear of rail vehicle wheels, and they expounded the influence of the wheelset coaxial wheel diameter difference on the creep force and wear power of the wheel. Liu et al. [12] studied the influence of the wheelset coaxial wheel diameter difference on a locomotive’s dynamic performance and wheel–rail contact, and they emphatically analyzed the influence of the wheelset coaxial wheel diameter difference on a locomotive’s dynamic performance and the wheelset coaxial wheel diameter difference on wheel–rail contact through a dynamic simulation and finite element calculation. Wang et al. [13] established a dynamic model by using SIMPACK software, predicted the trend of wheel wear combined with the Archard wear model and compared and analyzed the influence of four different types of wheelset coaxial wheel diameter differences on wheel flange wear. Ding et al. [14,15] took a C80 truck as an example to analyze the influence of the deflection angle and wheelset coaxial wheel diameter difference on wheel wear. The results showed that the wheelset coaxial wheel diameter difference mainly affects the lateral movement of the wheelset, easily causing wheel offset and increasing the wear rate of the wheelset tread. Based on the vehicle model, Li et al. [16] analyzed the dynamic performance of a tread with different degrees of wear, and the results showed that changes in the wheel shape and wheel diameter affect wheel–rail contact, leading to the deterioration of the vehicle’s dynamic performance. Ma et al. [17] studied the influence of the wheelset coaxial wheel diameter difference on wheels and rails under an inert state and electric braking state, and the results showed that the wheelset coaxial wheel diameter difference changes the motion state of the wheelset, aggravates the wear between the wheel and the rail and, at the same time, also causes the eccentric wear of the wheelset. Wang et al. [18] studied the influence of the wheelset coaxial wheel diameter difference on the wheel tread wear rate, depth and rolling fatigue, and the results showed that the wheelset coaxial wheel diameter difference leads to wheelset offset and tread wear rate acceleration but also causes wheelset offset, expanding the rolling fatigue area of the wheelset and reducing the service life of the wheelset. Based on the research results of scholars on the wheelset coaxial wheel diameter difference, it can be concluded that the wheelset coaxial wheel diameter difference causes the lateral movement of the wheelset, changes the wheel–rail contact area, causes the partial wear of the wheelset, induces the formation of grooved treads and affects the vehicle’s ride, safety and other aspects of dynamic performance. In addition, the wheelset coaxial wheel diameter difference also promotes the initiation of cracks on the surface of the wheelset, reduces the service life of the wheelset and increases the operating cost of the vehicle.

In terms of the impact of the wheelset coaxial wheel diameter difference on a vehicle’s dynamic performance, many scholars have provided many ideas and conducted much research [19,20,21,22,23,24]. Although the academic world has conducted extensive and in-depth research on the wheelset coaxial wheel diameter difference, there is still a large gap in monitoring the wheelset coaxial wheel diameter difference through a trackside monitoring system. Especially through a lateral displacement measurement method. The relationship between the wheelset coaxial wheel diameter difference and the lateral displacement of the wheelset can be determined through the change in the lateral displacement of the wheelset under the condition of the rail diameter difference. Through this relationship, the difficulty of monitoring the wheelset coaxial wheel diameter difference of the trackside system can be greatly simplified. Therefore, based on research on the lateral displacement of the trackside system under the condition of the wheelset coaxial wheel diameter difference, online monitoring of the wheelset coaxial wheel diameter difference is worth elaboration.

In this paper, the response of the lateral displacement of a heavy-haul freight car under the condition of the wheelset coaxial wheel diameter difference is studied, and the mapping relationship of lateral displacement under the condition of the wheelset coaxial wheel diameter difference is derived to realize the effect of detecting the wheelset coaxial diameter difference through a trackside detection system. The analysis results are verified via combination with simulation and test data. In the rest of the paper, the second section mainly introduces the detection method of the trackside wheelset lateral displacement, the Section 3.1 presents the dynamic model construction of the vehicle–track coupling system based on the trackside detection method, the Section 3.2 presents the application of a field test to verify the dynamic model, and the Section 4.1 analyzes the mapping relationship between the lateral displacement of the wheelset and the difference between the wheelset and wheel diameter. In Section 4.2 the proposed mapping relationship is applied to the trackside system, and the reliability of the research method is verified via a large amount of data detection.

## 2. Material and Methods

**Figure 2:**
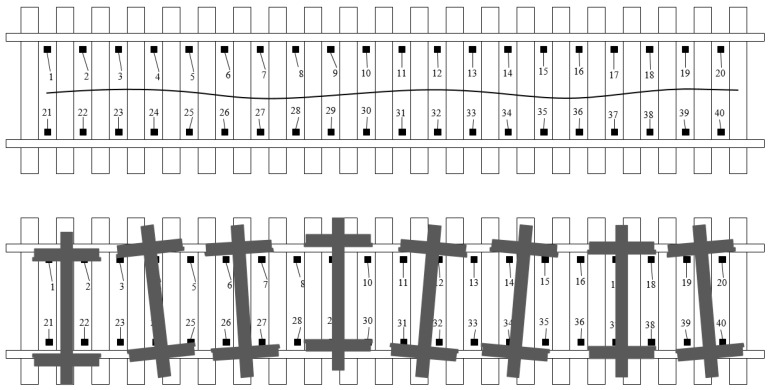
Schematic diagram of lateral displacement detection method.

By testing the lateral displacement of the wheelset, the running state of the vehicle can be monitored. The eddy current sensor can be used to test the lateral position of the wheelset with high precision. Moreover, compared with the laser displacement sensor, the eddy current sensor has the characteristics of less interference from other nonmetallic objects, a strong anti-fouling ability and less influence from the wheel contour. Figure 2 shows the layout plan of the proposed displacement sensor. The black dots marked with orderly numbers in the figure are eddy current sensors, which need to be installed between two sleepers with special mechanical tools. According to the calculation that the span of each sleeper is 600 mm, the system arranges 20 rail span sensors with a length of 12 m. The system chooses to install sensors on both sides of the same track to ensure that the lateral displacement data collected by the system can be tested to obtain the inside distance of the wheelset and the yaw angle of the wheel.

**Figure 3:**
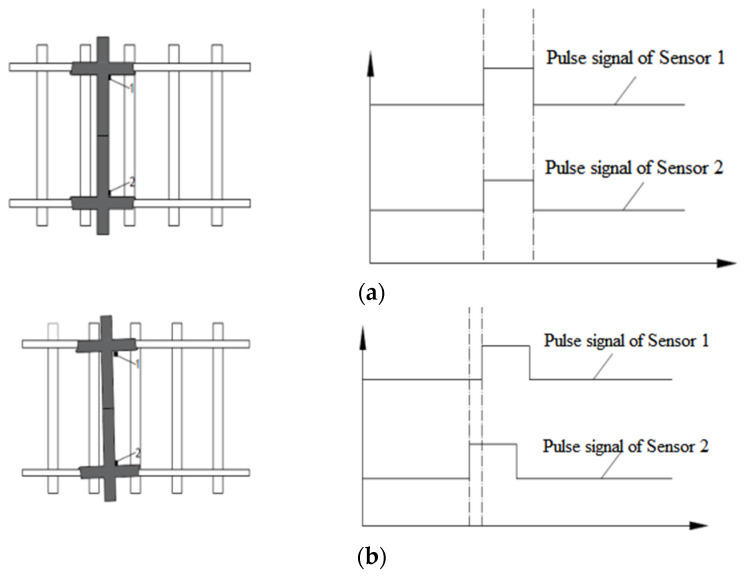
Test principle of wheelset lateral movement and yaw angle (yaw angle is not present (**a**), yaw angle is present (**b**)).

Figure 3 shows the systematic test method of the wheelset yaw angle. When there is no wheelset yaw angle, the start and end time of the step signals collected by the displacement sensors on both sides should be in the same position (as shown in Figure 3a). When the wheelset yaw angle occurs, the start/end time of the step signal collected by the left and right sensors is delayed in time (as shown in Figure 3b). According to the wheel speed *v,* the time difference *δt* and the inner distance of the wheelset *l*, the specific value of the yaw angle of the wheelset can be measured with the conversion formula *θ* = arctan[(*v* × *δt*)/*l*].

The position signal collected by each individual eddy current sensor on each side can be put into the data processing system for calculation and analysis, and the highest point of the displacement curve measured is taken, multiplied by the calibration coefficient and carried out continuously in spatial order. Then, the wheel displacement curve in space can be obtained when the vehicle passes the test position. The position curve of the left and right wheels can be used to obtain the alignment of the detected wheelset and to provide data support for the inversion of the wheelset coaxial wheel diameter difference.

**Figure 4:**
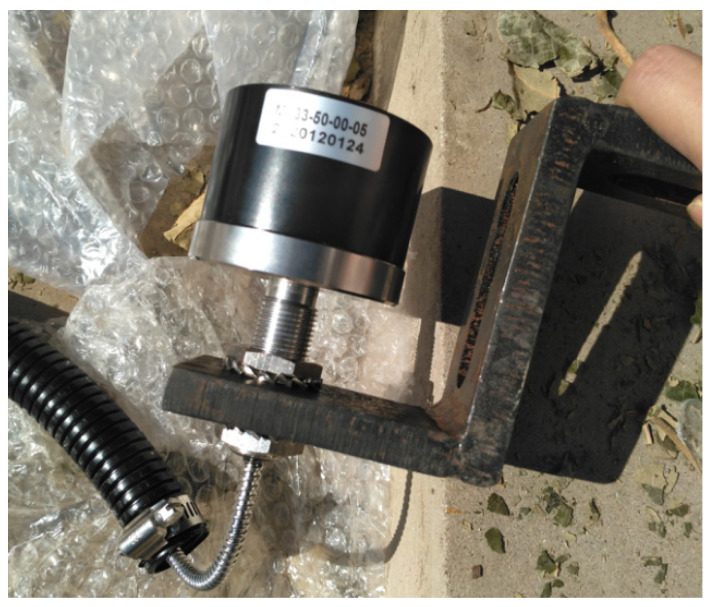
Eddy current sensor for testing system.

Based on the measurement of the wheelset lateral displacement, a ground non-contact measurement method is adopted, and the CDS-55VM10 eddy current sensor (Figure 4) is used. An eddy current sensor is a non-contact sensor that can be used statically and dynamically and that can measure the distance between the measured metal conductor and the probe surface with high linearity and high resolution. It is a non-contact linearized measurement tool. Table 1 lists the detailed parameters of the eddy current sensor used in this paper.

**Figure 5:**
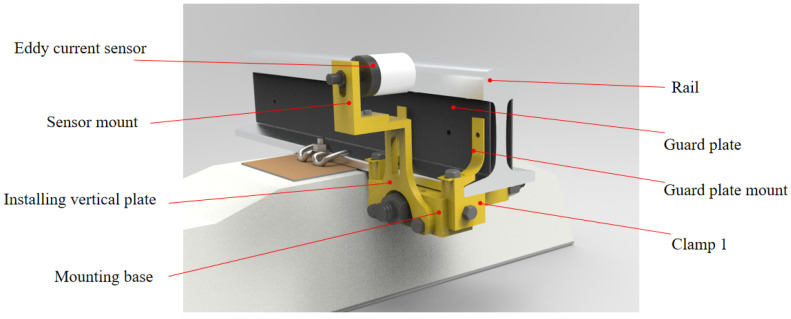
Eddy current sensor and its support device.

The eddy current sensor is installed between sleepers using dedicated tools. Figure 5 shows the dedicated tools for installing the eddy current sensor and the installation effect. The device is composed of fixture 1, fixture 2, a mounting base, a mounting plate and a sensor support. During installation, fixture 1, fixture 2 and the installation base are fixed on the bottom of the rail, and then the mounting plate is installed on the mounting base and connected through two M12 threaded holes on the installation base. The bottom of the sensor support and the top platform of the vertical plate are fastened with two M6 bolts and nuts. The eddy current sensor is installed on the long hole of the sensor mounting seat through the nut screw on the back, the installation height of the eddy current sensor is adjusted through the position of the long hole, and the lateral displacement between the eddy current sensor and the rail is adjusted through the screw on the back of the eddy current sensor.

**Figure 6:**
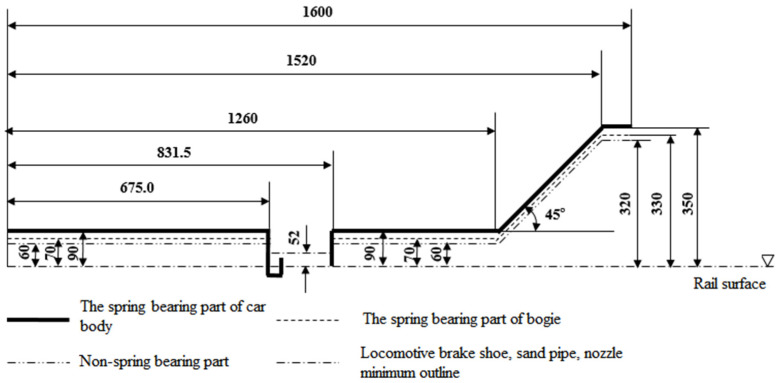
Bottom limit for rolling stock.

**Figure 7:**
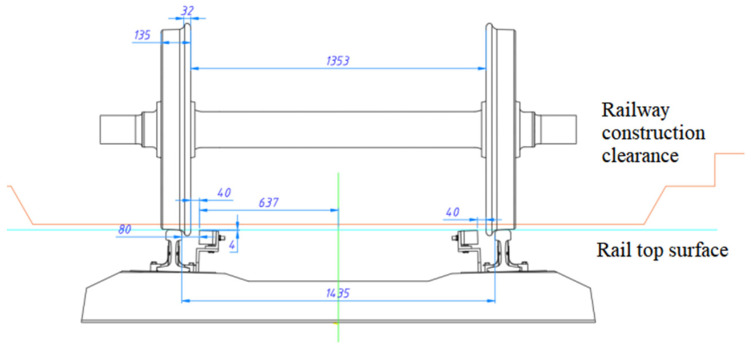
Corresponding position of truck wheelset and eddy current sensor.

To ensure that the device does not interfere with the safety of vehicle operation, the sensor is installed 4 mm below the rail surface, and the lateral distance of the sensor test end face is 30 mm from the wheel back so as to ensure that the sensor is always outside the bottom limit of the rail of the vehicle (Figure 6) and does not affect the safety of the locomotive and vehicle operation. The corresponding position of the wheel and sensor installation is shown in Figure 7.

Two eddy current displacement sensors between each sleeper serve as an expandable test unit, and all sensors are connected to the data acquisition instrument and control equipment through shielded cables. The boot sensor and vehicle number identifier should be installed in the coming direction of the remote side of the test equipment. When the system senses that there is a wheel approaching, the control device automatically triggers to open the acquisition instrument. Two eddy current displacement sensors in each test unit respectively output pulse signals. When the wheelset yaw angle does not occur (Figure 3a), pulse signals output by two eddy current displacement sensors in the same test unit have the same phase. The amplitude of pulses on both sides is taken as the lateral movement of the current wheel at the test position. When the wheelset yaw angle occurs (Figure 3b), the pulse signals output by two eddy current displacement sensors in the same test unit have a phase difference. The phase difference of the pulse combined with the speed can output the yaw angle of the wheelset. When each wheelset passes through each group of test units, the wheelset lateral movement and yaw angle are recorded so as to obtain the wheelset hunting movement frequency and hunting movement amplitude. This method uses a small number of sensors and equipment to realize the effective monitoring of the vehicle hunting movement amplitude, which has a good economy effect.

## 3. Theory and Calculation

### 3.1. Vehicle–Track Coupling Dynamics Model Based on Trackside Detection Method

**Figure 8:**
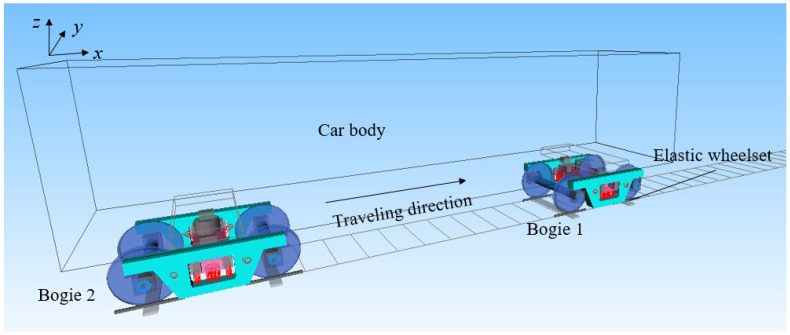

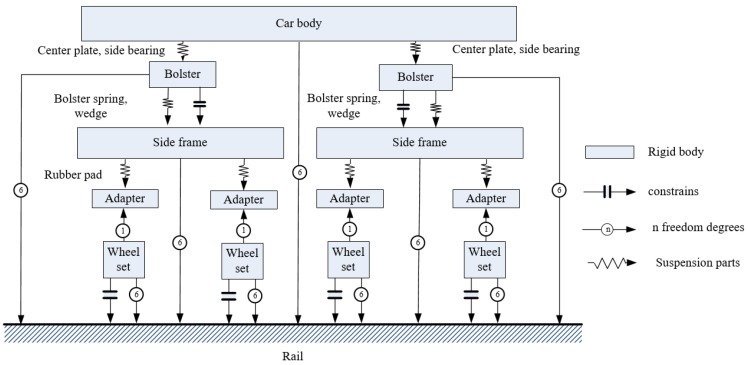
Simulation model and topological structure diagram of C80 wagon.

A freight vehicle system is a complex multi-body dynamic system with many nonlinear factors, such as nonlinear wheel–rail interactions and friction dampers. The characteristics of the vehicle hunting motion and the trajectory of wheelsets can be obtained through dynamic simulations. The multi-body dynamics software Simpack is used for the dynamic modeling of type C80 heavy-haul freight cars. During the simulation process, two different wheel tread states are selected, as shown in Figure 8. One is the wheel’s original state with a type LM tread profile, and the other one is the wheel’s serious wear state, with a wear depth of 1.19 mm in a running circle (*x* = 0). The simulation model and topological structure are as shown in Figure 8. The vehicle adopts a type K6 three-piece bogie, the wheelset is considered as an elastomer, the car body is considered as a rigid body, the influence of the onboard equipment vibration is not considered, the rail support is a discrete support, the under-sleeper support and the stiffness of the rail fasteners are taken into account, and the nonlinear characteristics of the elastic suspension of the truck are considered. The entire vehicle model contains 90 degrees of freedom, 1 body, 4 side frames, 2 bolsters that all have 6 DOF (3 moving DOF and 3 rotating DOF) and 8 bearing saddles, with 1 vertical DOF each. In addition, for 4 wheelsets, each has 6 DOF (3 moving DOF and 3 rotating DOF), 1 bending DOF and a torsional elastic DOF. For 8 wedges, there is 1 vertical DOF each. The vehicle model takes the nonlinear wheel–rail contact geometry into consideration. Some important parameters of the vehicle are shown in Table 2.

**Figure 9:**
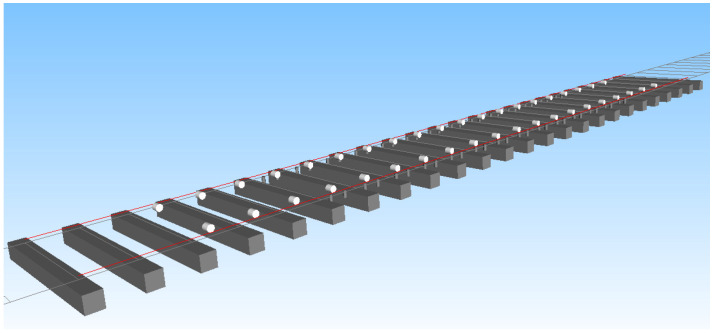
Track model with eddy current displacement sensors.

**Figure 10:**
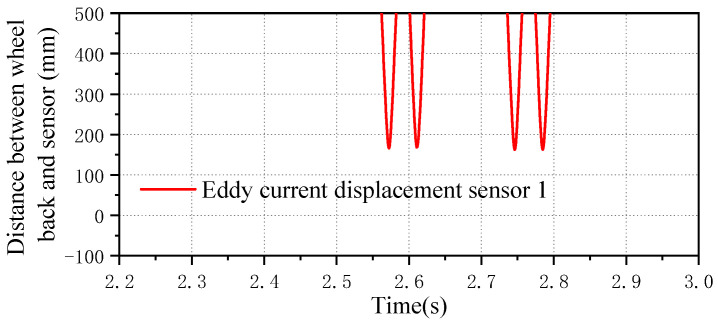
Wheel passing signal of simulated sensors.

The simulation model needs to achieve an effect close to field detection so as to achieve a better data verification effect in the later period. The sensors to be installed in the on-site trackside detection equipment are eddy current sensors, which are arranged according to the detection scheme. In other words, 20 rail spans are arranged on the left and right rail sides, with a total of 40 sensors. For the detection of the wheelset lateral displacement, the displacement sensor in Simpack is used for testing. Figure 9 shows the simulation interface layout effect of the lateral displacement sensor. Figure 10 shows the detection effect of the simulation sensor. When the wheel rolls past the measurement point, the test signal gradually decreases to the minimum value, which is the distance between the detected wheel back and the sensor. It can see that there are four minimum values in the figure, which in turn are the lateral displacement values of the first wheelset to the fourth wheelset. When the displacement value reaches the minimum value, it means that the wheelset just passes the test point at this time.

### 3.2. Model Verification

**Figure 11:**
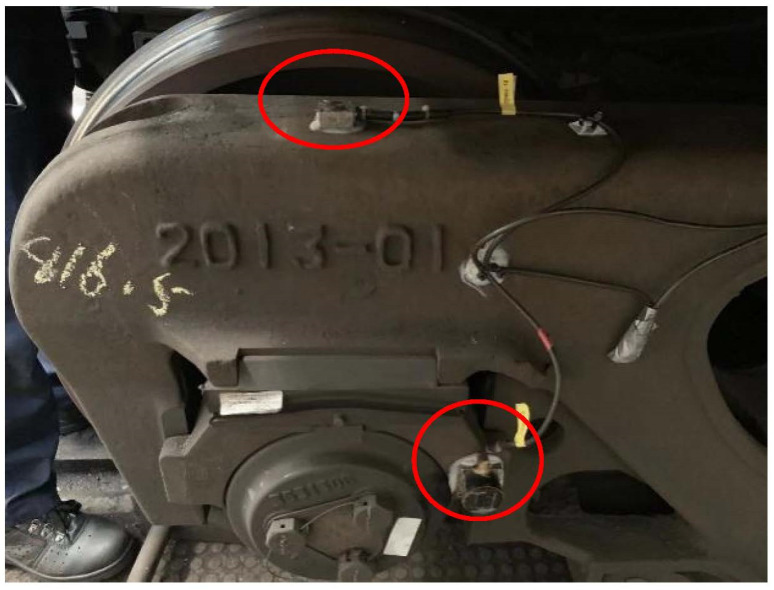
Installation position of test sensor.

The verification of the vehicle’s system model is different from that of a trackside signal acquisition system. As the main research object of this paper, whether the vehicle can reflect the actual operation of the vehicle is very important for further research. This section mainly uses a field loading test to verify the simulation model established above. Specifically, it verifies the lateral vibration acceleration of the left front segment of the freight car bogie frame in the empty vehicle state, including the time domain state and frequency domain state of vibration acceleration. Figure 11 shows the installation position of the test sensor. The measuring point is the end of the left-side frame of the front bogie.

**Figure 12:**
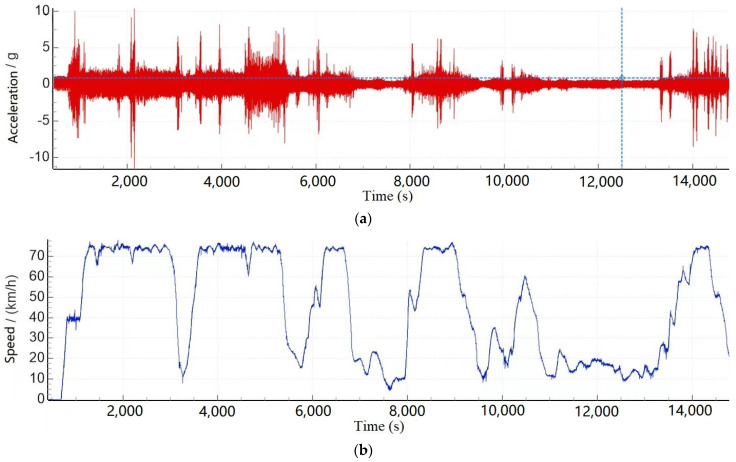
Time domain diagram of vibration acceleration of the left front bogie frame and its corresponding speed diagram. ((**a**) Acceleration signal, (**b**) speed).

The field test was on the normal operation line, and the test section was Huanghua Port, Shenchinan. The vehicle was running in the normal operation state. Figure 12a shows the time domain signal collected by the lateral vibration acceleration sensor at the end of the left-side frame, and Figure 12b shows the running speed of the corresponding vehicle. It can be seen that the maximum speed of the vehicle in the test is about 75 km/h, and part of the high-frequency excitation occurs during the test.

**Figure 13:**
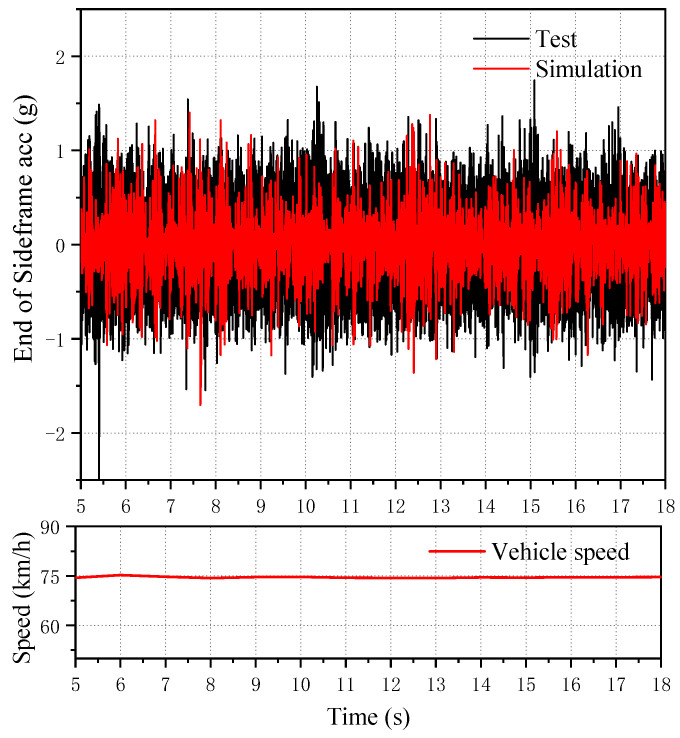
Comparison of the vibration acceleration (partial interception) of the left front end of the front bogie side frame and the time domain diagram of the same test location of the simulation model.

**Figure 14:**
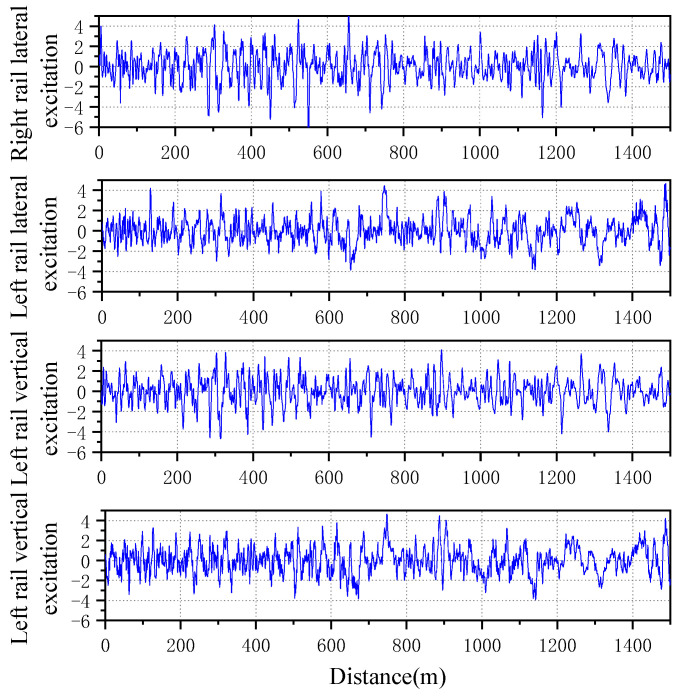
Selected Shenhua orbital excitation spectrum.

In the test data of the test vehicle, test data of about 20 s at a speed of 75 km/h were intercepted for data comparison, and 600 Hz low-pass filtering was performed to avoid the influence of high-frequency excitation on the data. The data are shown in a black solid line in Figure 13, and the following figure is the speed corresponding to the test data. The corresponding speed of the simulation model was set as 75 km/h, and the wheel tread measured on site was used for simulation. The Shenhua spectrum was selected as the track excitation spectrum, and the longitudinal, lateral and vertical excitation conditions are shown in Figure 14.

Figure 13 shows the lateral vibration acceleration of the end of the simulated side frame. It can be seen that, at this time, the simulation results are basically consistent with the measured data in amplitude, and the vibration amplitude is basically around 1 g. The test data are slightly larger than the simulation data in amplitude but are basically consistent.

**Figure 15:**
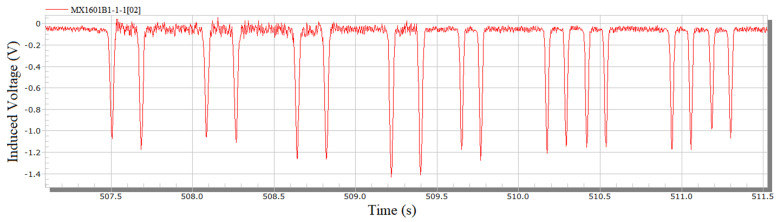
Field lateral displacement original signal.

**Figure 16:**
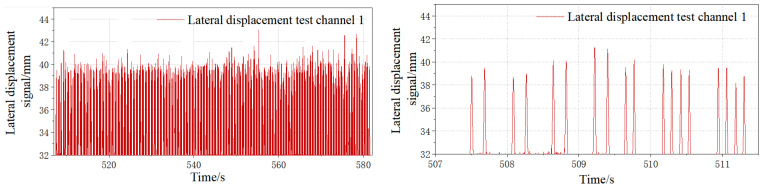
Field lateral displacement conversion signal.

Figure 15 shows the original signal of the wheelset lateral displacement collected on site. The passing signals of each wheel can be well collected by the sensor without any influence, such as signal burr, and the passing vehicles and wheels can be clearly seen. By multiplying the calibration coefficient and substituting in the initial displacement value, the lateral displacement signal, as shown in Figure 16, can be obtained.

As can be seen from Figure 16, lateral displacement signals on site can identify each wheel signal and distinguish the vehicle sequence of the wheel. From the signal results, all wheelsets passing through the first sensor are located near the center of the track. The lateral displacement signal has only one effective data point at the peak position of the signal, i.e., the absolute lateral position signal of the wheel collected.

**Figure 17:**
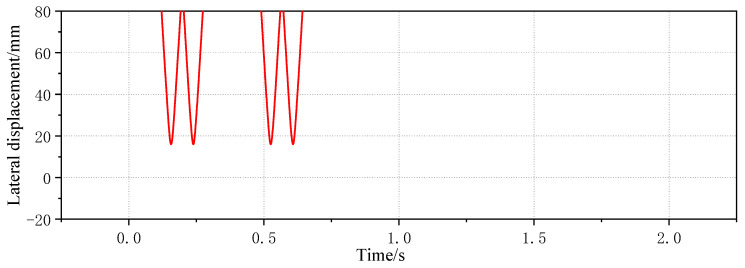
Lateral displacement signal from simulation.

The simulated lateral displacement signal is shown in Figure 17. It can be seen that the sensor can obtain the wheel displacement signal by sampling, and the vehicle speed can be calculated from the time interval of the effective displacement sampling of different wheels. Although the simulated lateral displacement signal is greatly reduced and the trough is the effective displacement data point, the sampling form is basically the same as that of the field testing system, and the signal results and effects are the same. Therefore, it can be considered that the detection effect of the simulation model is the same as that of the field measurement, which verifies the reliability of the trackside detection system of the simulation model.

## 4. Results

### 4.1. Mapping Relationship between Wheelset Coaxial Wheel Diameter Difference and Trackside Lateral Displacement (With and Without Excitation)

**Figure 18:**
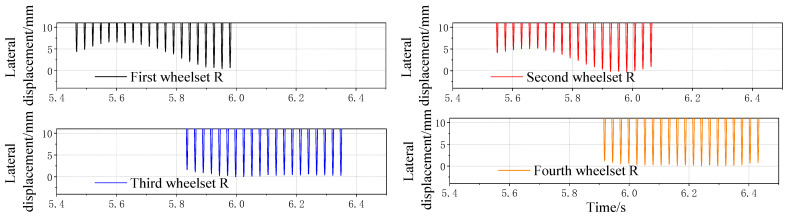
Time domain diagram of four wheelsets’ lateral displacement detected with simulation trackside system with 1 mm wheelset coaxial wheel diameter difference.

**Figure 19:**
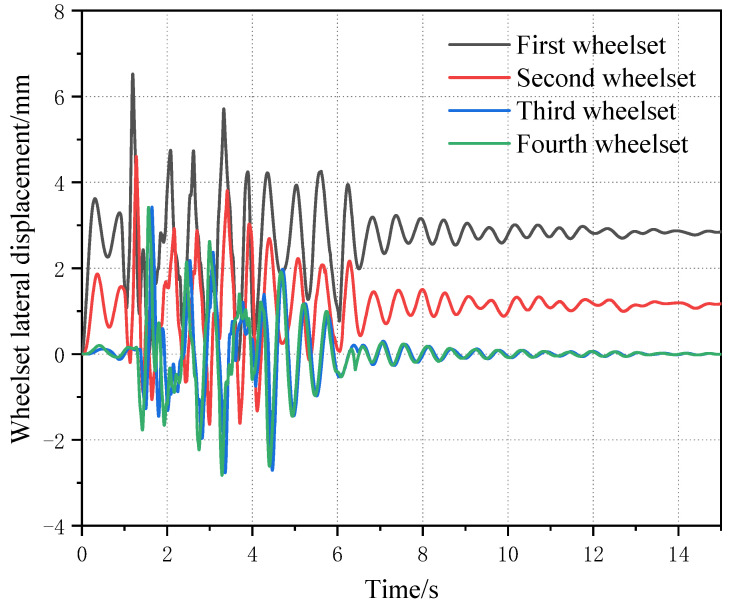
Time domain diagram of four wheelsets’ lateral displacement with 1 mm wheelset coaxial wheel diameter difference.

The relationship between the wheelset coaxial wheel diameter difference and lateral displacement was analyzed, and the working condition of the wheelset coaxial wheel diameter difference was set as 1–5 mm, with the right wheel diameter of one wheelset decreasing and the other wheel diameter unchanged. Only the first wheelset possesses a diameter difference. Figure 18 and Figure 19 show the time domain diagram of the lateral displacement of four wheelsets of a vehicle with a 1 mm wheelset coaxial wheel diameter difference at a speed of 60 km/h. The right side of the running direction of the vehicle is the positive direction of the lateral movement of the vehicle. It can be seen from the figure that the difference in the wheel diameter of one wheelset causes the wheelset to run on one side. It can be seen from the results that the difference of the wheelset diameter of one wheelset has a greater impact on the other wheelset on the same bogie, but it has little impact on different bogie wheelsets of the same vehicle.

**Figure 20:**
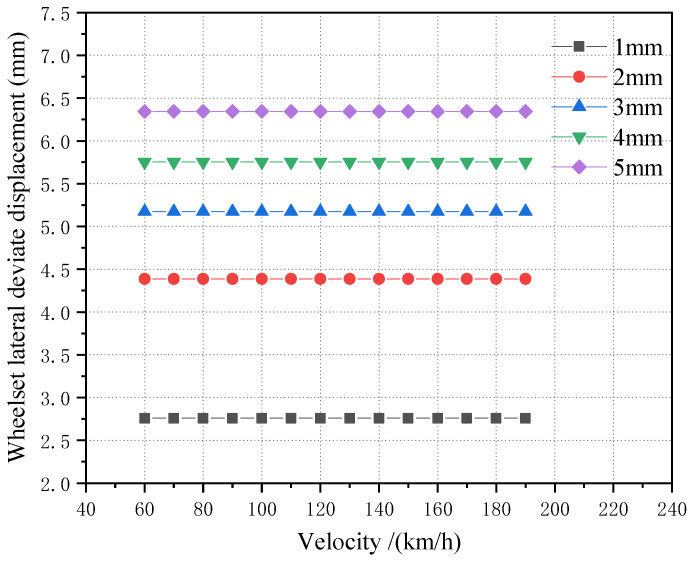
LM tread state, the influence of different speeds on the offset under the condition of 1–5 mm wheelset coaxial wheel diameter difference.

**Figure 21:**
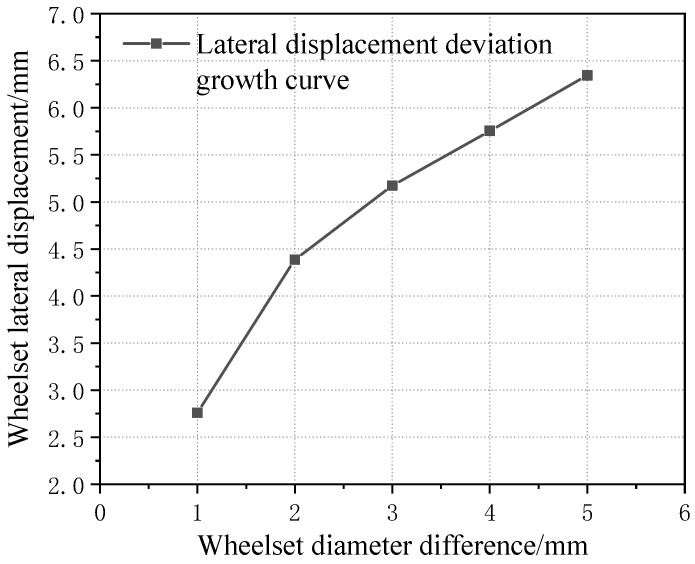
Curve of variation between wheelset lateral displacement and wheelset coaxial wheel diameter difference.

The lateral displacement of the first wheelset under a 1–5 mm wheelset coaxial wheel diameter difference with an LM initial tread is compared, as shown in Figure 20. It can be seen that, in the case of hunting movement, the value of the wheel’s lateral displacement does not change with the increase in vehicle speed, but with the increase in the wheelset coaxial wheel diameter difference, the value of the first wheelset’s lateral displacement during operation gradually increases. Moreover, the growth rate slows down, as shown in Figure 21.

**Figure 22:**
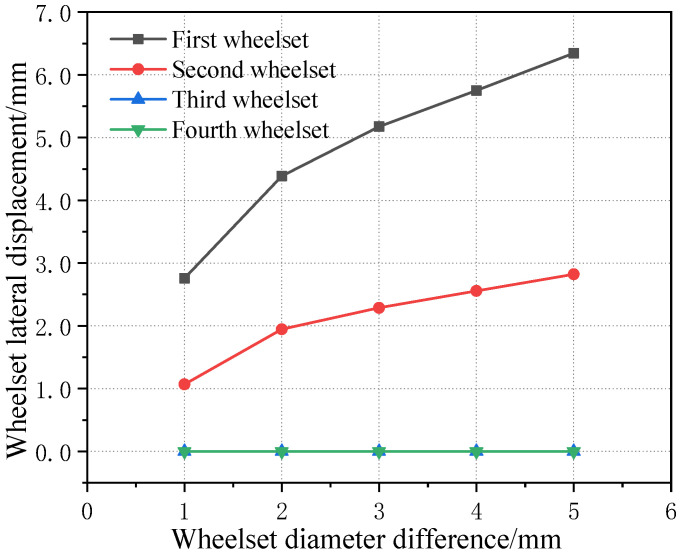
Curves of four wheelsets changing with wheelset coaxial wheel diameter difference of the same vehicle.

The four wheelsets of the same vehicle are placed under the same picture for comparison, as can be seen from Figure 22, in which the first wheelset’s and the second wheelset’s lateral displacement curve lines are basic same. For the scale, the lateral displacement of the first wheelset is the largest, and the two wheelsets of another bogie of the same vehicle also have an operating centerline deviation due to the wheelset coaxial wheel diameter difference of the first round pair. However, the influence is small.

**Figure 23:**
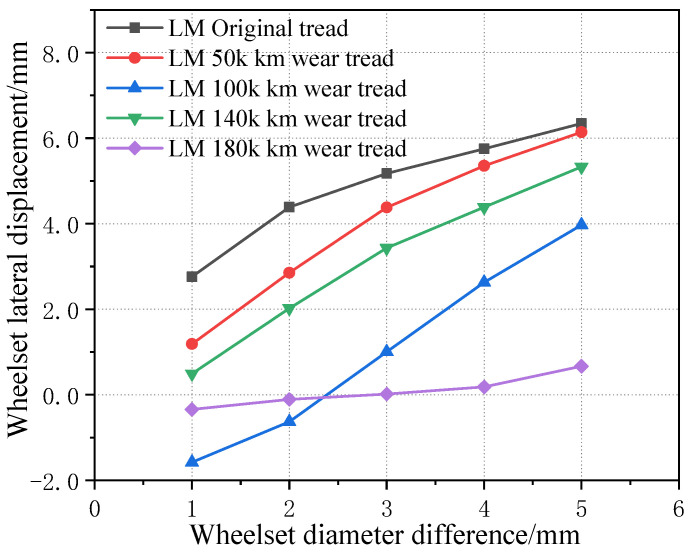
Influence of wheelset coaxial wheel diameter difference on lateral offset of wheelset under five tread conditions.

**Figure 24:**
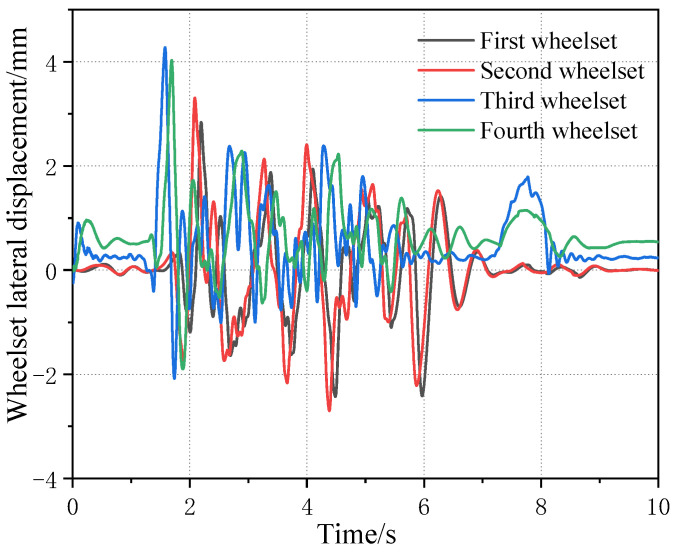
Time domain diagram of lateral displacement of four wheelsets at a speed of 60 km/h with 4 mm wheelset coaxial wheel diameter difference on LM180 k km worn tread (wheelset axial diameter difference occurs in first wheelset).

Figure 23 shows the influence curve of the wheelset coaxial wheel diameter difference on the wheelset lateral displacement under the condition of an LM initial tread and four worn wheel treads in one rotational repair cycle. It can be seen that, with the increase in the wheelset coaxial wheel diameter difference, the lateral displacement offset of the wheelset under all tread conditions increases, and the growth rate decreases gradually. However, in the middle stage of tread wear, i.e., a 50 k km–140 k km worn tread, the difference in the lateral offset of the wheelsets is not obvious under the three kinds of tread conditions because the equivalent conicity of the tread is relatively close. When the wheel is worn to the later stage, due to the increase in the equivalent conicity of the wheel, the lateral displacement of the wheelset decreases a lot and is basically within a 0.5 mm offset, which is caused by a large lateral component of gravity on the wheel when the equivalent conicity is large. At the same time, because the equivalent conicity of the tread is large, the linear critical speed of the wheelsets’ hunting instability also decreases relatively so, resulting in the phenomenon of instability at low speeds (as shown in Figure 24), which further affects the measurement results of the lateral offset.

**Figure 25:**
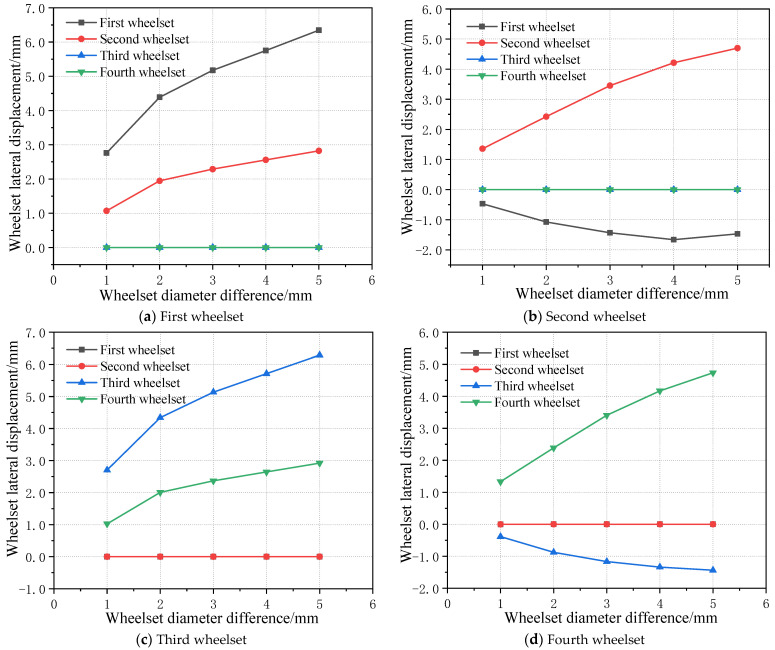
Influence of wheelset coaxial wheel diameter difference at different positions on wheelset lateral movement.

When the wheelset coaxial wheel diameter difference occurs in a non-guiding wheelset, the lateral movement state of each wheelset of the vehicle may change. Therefore, the influence of the wheelset coaxial wheel diameter difference at different wheelset positions on the wheelset lateral movement was simulated and analyzed. Figure 25 shows the lateral offset of each wheelset when the wheelset coaxial wheel diameter difference occurs in four wheelsets of the same vehicle. It can be seen that, when the wheelset coaxial wheel diameter difference appears, it has a greater impact on another wheelset of the same bogie and a smaller impact on the wheelset of different bogies of the same vehicle. At the same time, it can be seen that, for the first bogie, the lateral offset of the first wheelset is always greater than that of the second wheelset regardless of whether the wheelset coaxial wheel diameter difference occurs in the first wheelset or the second wheelset. However, when the wheelset coaxial wheel diameter difference occurs in the second wheelset, the lateral offset amplitude of the second wheelset is smaller than that of the first wheelset, which is because the first wheelset is the guiding wheelset.

When the wheelset coaxial wheel diameter difference appears on the second wheelset, the lateral displacement of the wheelset has an obvious change, and when the wheelset offset value of the first bogie is negative, i.e., the first bogie appears to run on the left side of the railway, at the same time with the increase in the speed, the bogie deviation to the left side of the operation of the deviation value continues to increase. Moreover, it can be seen that, when the wheelset coaxial wheel diameter difference occurs in the third wheelset, the variation rule of the offset of the third and fourth wheelset is basically the same as that of the first and second wheelset when the wheelset coaxial wheel diameter difference is located in the second wheelset, both of which show that, with the increase in the wheelset coaxial wheel diameter difference, the offset value of the third and forth wheelset increases gradually at a decreasing rate. Moreover, the offset value of the third and fourth wheelset is basically the same. When the wheelset coaxial wheel diameter difference is small, the offset value of the wheelset with the wheelset coaxial wheel diameter difference is larger than that of the normal wheelset. However, with the increase in the wheelset coaxial wheel diameter difference, the lateral offset value of the normal wheelset gradually exceeds the wheelset with the wheelset coaxial wheel diameter difference fault. However, when the wheelset coaxial wheel diameter difference appears in the fourth round, the offset of all wheelsets is the smallest in all working conditions, and its maximum offset value is 2.9 mm, which is nearly twice as small as 5.6 mm in the first wheelset. It can be seen that, at this time, the influence of the wheelset coaxial wheel diameter difference on the running state of the vehicle is minimal. Therefore, it can be predicted that the influence of the location of the wheelset coaxial wheel diameter difference on the vehicle dynamics is as follows: guide wheel > non-guide wheel > rear wheel, which is basically consistent with the rule of wheel tread wear at different positions.

Polynomial fitting is applied to fit the relationship between the wheelset coaxial wheel diameter difference and the wheelset lateral displacement when the wheelset coaxial wheel diameter difference occurs at different wheel positions, and the mapping relationship between the wheelset coaxial wheel diameter difference and the lateral displacement can be obtained. Based on the above analysis, the mapping relationship can be summarized as follows.

Let the lateral displacement be *y* and the wheelset coaxial wheel diameter difference be *x*. For the wheelset coaxial wheel diameter difference of the first wheelset, the fitting relation between the lateral displacement value of each wheelset and the wheelset coaxial wheel diameter difference value is as follows:

First wheelset:

*y* = −0.1629*x*^2^ + 1.83054*x* + 1.1834 (1)

Second wheelset:

*y* = −0.0925*x*^2^ + 0.9669*x* + 0.2544 (2)

Third wheelset:

*y* = 1.68 × 10^−7^*x*^2^ + 6.782 × 10^−7^*x* − 1.146 × 10^−6^ (3)

Fourth wheelset:

*y* = 4.87857 × 10^−8^*x*^2^ + 1.1054 × 10^−6^*x* − 1.519 × 10^−6^ (4)

By synthesizing the working conditions of four wheelsets with the wheelset coaxial wheel diameter difference and summarizing, in the LM initial tread state, the mapping relationship between the wheelset lateral movement and wheelset coaxial wheel diameter difference can be obtained when the wheelset coaxial wheel diameter difference occurs in each wheelset, as shown in Table 3.

The existence of a wheelset coaxial wheel diameter difference leads to a situation in which the vehicle runs on one side, and a situation in which vehicle off-loading may also lead to a deviation in the vehicle. Therefore, it is also necessary to analyze the deviation in the vehicle caused by off-load operations so as to eliminate the influence of off-loading on wheelset coaxial wheel diameter difference monitoring.

**Figure 26:**
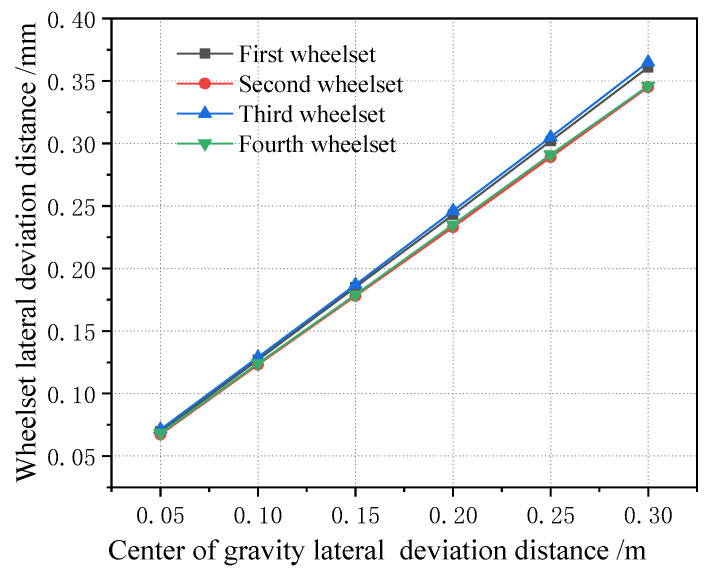
Change in each wheelset lateral displacement with lateral deviation in the car body center of gravity when the longitudinal gravity center offset is 0 m.

**Figure 27:**
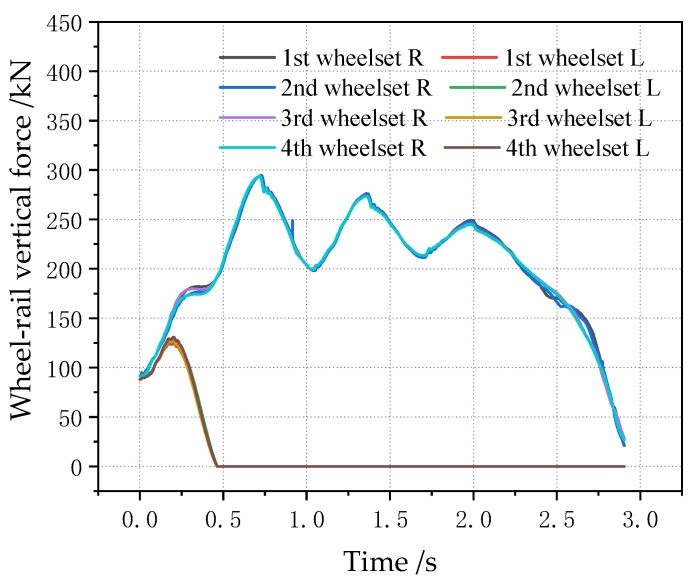
Change in wheel–rail force of each wheel with a lateral deviation in the center of gravity of 0.5 m.

The off-loading condition of the vehicle is analyzed, and the off-loading condition of the vehicle generally occurs to the left, right, front and rear. Therefore, the working condition is set as the full load condition for the vehicle (i.e., the load is 80 t), the off-loading range of the center of gravity in the positive x direction is 0~2 m, the off-loading range in the positive y direction is 0~0.3 m, the step length in the y direction is 0.05 m, the step length in the x direction is 0.25 m, and the simulation calculation is carried out. Figure 26 shows the change in four wheelsets of a vehicle when the center of gravity is located in the longitudinal center of the car body. It can be seen that, with the lateral deviation in the center of gravity, the lateral displacement of the four wheelsets of the vehicle gradually increases, showing a linear growth change rule. However, the lateral displacement of the four wheelsets of the vehicle is not large, with a magnitude within 0.3 mm, which is only one tenth of the variation range of the wheelset coaxial wheel diameter difference. At the same time, it can be seen that, when the center of gravity is located in the longitudinal center of the vehicle body, the offset position of each wheelset has a small difference, and the lateral displacement deviation is within 0.03 mm, which can be basically considered to be in the same lateral position. It is worth noting that, when the lateral deviation in the displacement of the center of gravity is 0.5 m, the vehicle rolls over. Figure 27 shows the time domain diagram of wheel and rail force changes. Therefore, when the lateral shift of the center of gravity of the vehicle body is 0.3 m, it is already in a serious off-load condition.

**Figure 28:**
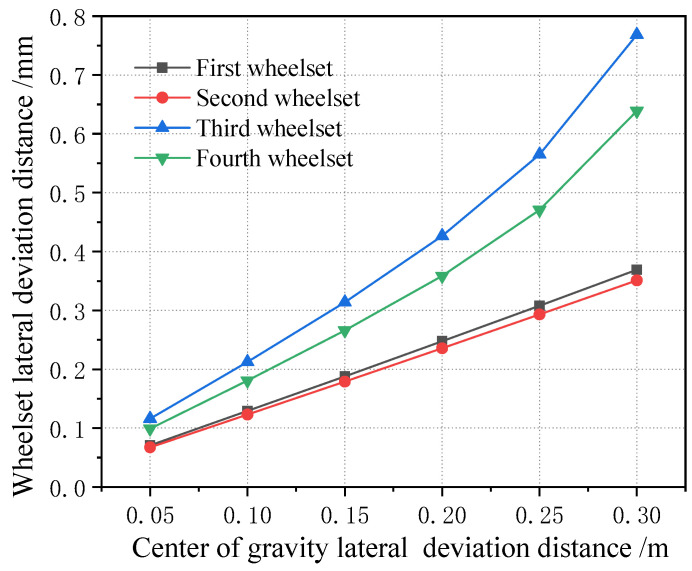
Changes in lateral displacement of each wheelset when the center of gravity is moved 2 m longitudinally forward.

**Figure 29:**
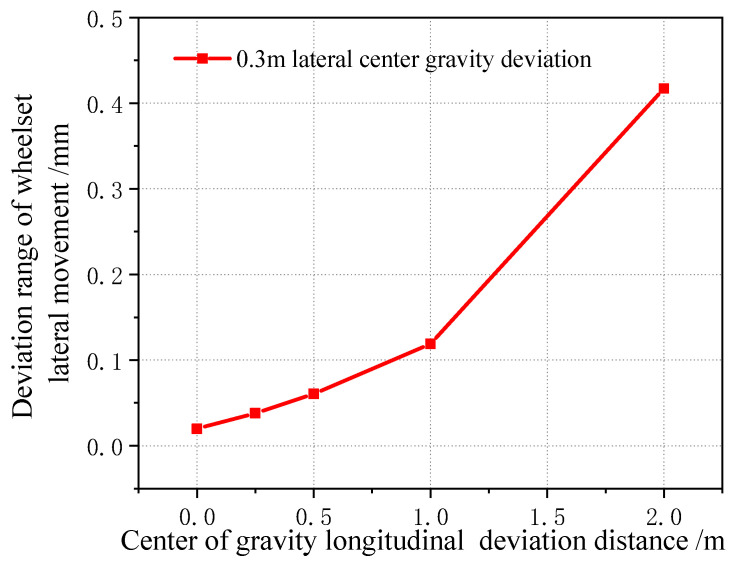
Variation range of wheelset lateral displacement with longitudinal center of gravity forward deviation.

**Figure 30:**
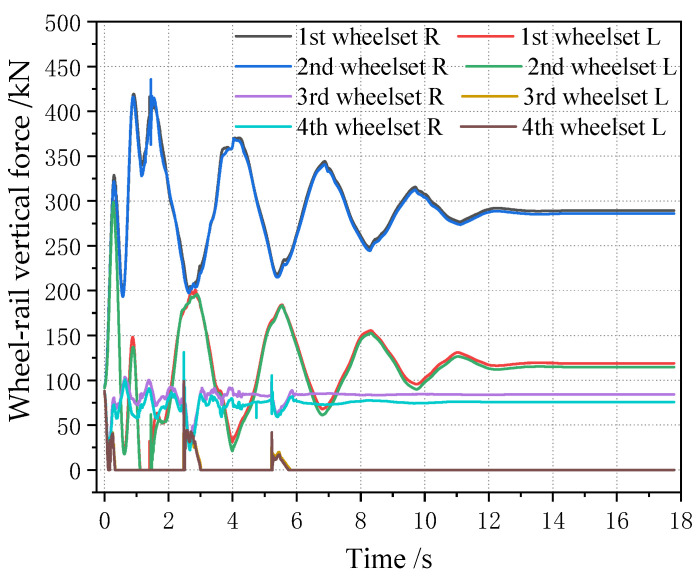
When the longitudinal center of gravity is offset forward by 3 m, the wheel–rail force of each wheel changes.

When the center of gravity is shifted along the longitudinal direction, the lateral displacement of the vehicle wheelset is changed, as shown in Figure 28 (longitudinal displacement of 2 m). It can be seen that, when the center of gravity is shifted along the longitudinal direction, the magnitude of the lateral displacement of the four wheelsets gradually increases, but the magnitude is still smaller than the wheelset coaxial wheel diameter difference, and the variation range is within 0.5 mm. Figure 29 shows the curve of the change in the four wheelsets of the vehicle along with the longitudinal deviation when the center of gravity of the vehicle is offset longitudinally. It can be seen that, with the increase in the longitudinal deviation, the lateral deviation in the four wheelsets of the vehicle gradually increases. It is worth noting that, when the lateral shift in the center of gravity displacement is 0.5 m, the vehicle rolls over. The time domain diagram of the wheel–rail force change is shown in Figure 30. Therefore, it can be seen that, when the longitudinal offset load of the vehicle’s center of gravity reaches 2 m, it is a serious offset load.

**Figure 31:**
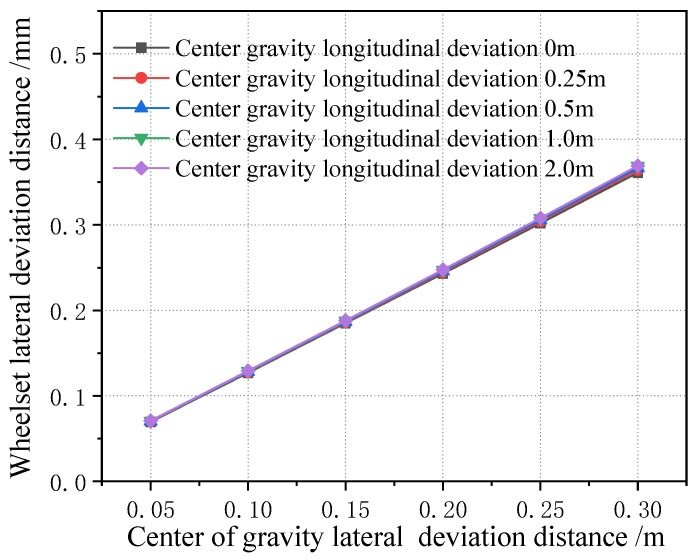
Variation in lateral movement of first wheelset under center of gravity longitudinal forward deviation.

Figure 31 shows how the lateral displacement of the first wheelset of a vehicle changes with the lateral deviation in the center of gravity under each longitudinal deviation in the center of gravity. It can be seen that, when the center of gravity is moved longitudinally, the variation range of the lateral displacement of the first wheelset of the vehicle is very small. Therefore, it can be proved that the influence of a vehicle’s off-load on the lateral displacement of the wheelset is small, which is about one tenth of the variation in the lateral displacement of the wheelset caused by the wheelset coaxial wheel diameter difference. Therefore, when the lateral displacement of the wheelset is used to identify the wheelset coaxial wheel diameter difference, the influence of the error caused by the vehicle’s offset load can be basically ignored. In addition, from the perspective of wheel–rail force analysis, the impact of the vehicle’s off-balance load state on the wheel–rail force is great, and the application of the wheel–rail force can realize the monitoring of the vehicle’s overbalanced load. As this paper only analyzes the wheelset coaxial wheel diameter difference and its monitoring and identification effect, it is not analyzed in detail here.

### 4.2. Test Verification

**Figure 32:**
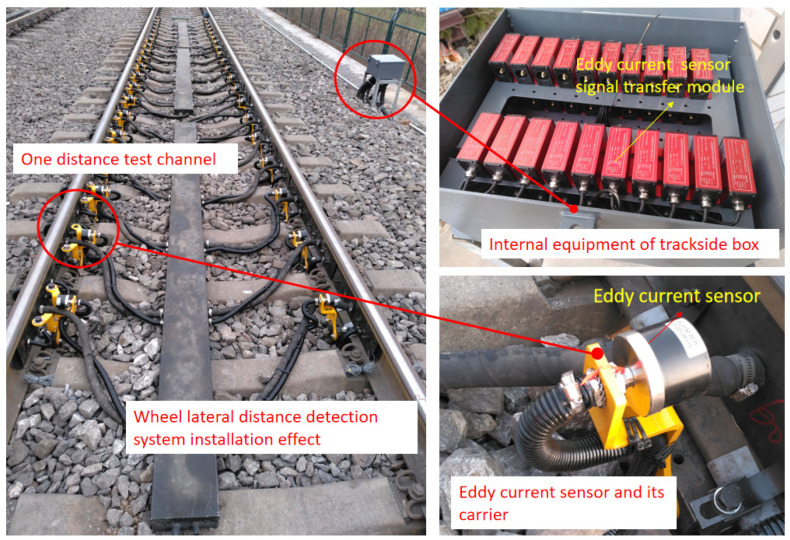
Installation of wheelset lateral distance detecting system.

The trackside system was installed on a heavy-duty coal transport line in China, and its installation effect is shown in Figure 32. The system was installed in a straight section, and the total length of the lateral instability monitoring area of the trackside detection system was 10.32 m. A total of 20 measuring points were arranged. At the system installation position, the passing vehicle speed interval was 40–80 km/h.

**Figure 33:**
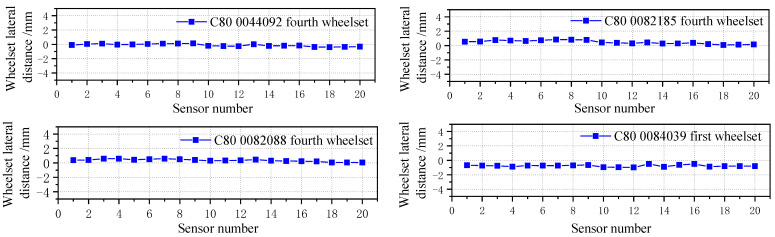
Normal wheelset lateral displacement signal.

The lateral displacement of the wheel can directly reflect the situation of the wheel tread’s abnormal wear or running to one side. As shown in Figure 33, it is the signal of the wheel displacement of the normal running wheel (without the wheelset coaxial wheel diameter difference or hunting instability).

**Figure 34:**
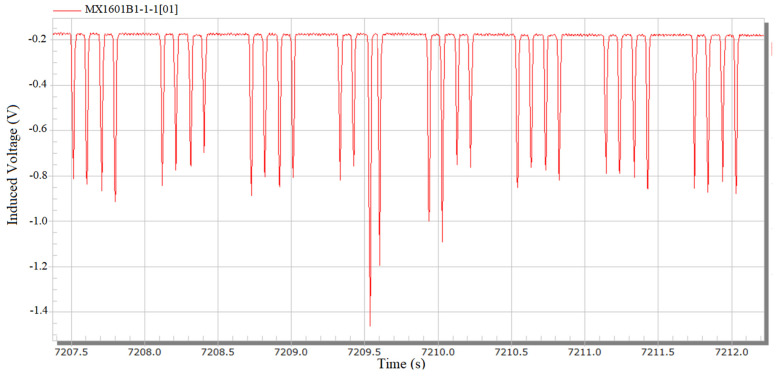
Abnormal wheelset lateral displacement original data.

The track of the wheel’s movement obtained with the signal of the wheel lateral displacement can be used to monitor the wheelset coaxial wheel diameter difference. As shown in Figure 34, the original data on wheel deviation were detected by the trackside detect system. It can be seen that the lateral displacement signals of the wheel were significantly different from those of other wheels, with an order of magnitude twice that of ordinary wheels. Moreover, four wheelsets belonging to the same vehicle all ran to one side in different degrees, for which it can be basically determined that the vehicle has a wheelset coaxial wheel diameter difference.

**Figure 35:**
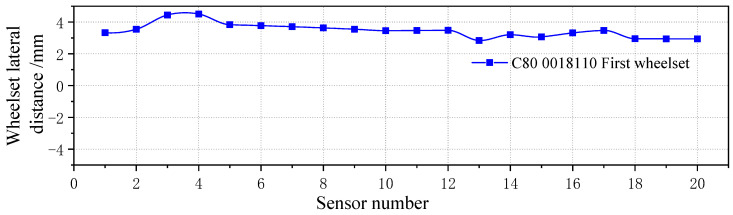
Lateral displacement of the deviated wheelset.

The vehicle was tracked, and the relevant vehicle information was finally located. Figure 35 shows the lateral movement trajectory of the measured wheelset. The wheel was that of a C80 freight car, number 0018110, and the wheelset was located on the first wheelset of the car. The measured wheelset deviated 4 mm from the track center, reaching the warning limit.

**Figure 36:**
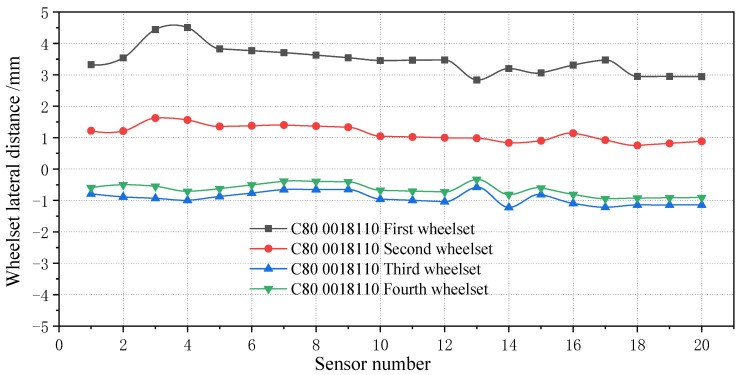
Lateral movement of four wheelsets of the same vehicle.

When the wheelset coaxial diameter difference occurs, it affects the displacement of the wheelset of the same bogie or even all the wheels of the same vehicle. Figure 36 shows the lateral deviation in the four wheelsets of the same vehicle.

**Figure 37:**
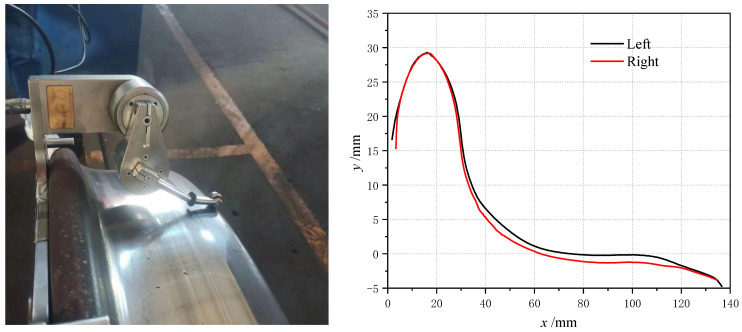
Tread profile and wheel diameter detection (**left**) and comparison of tread profiles (**right**).

During operation, the wheelset deviates 4 mm from the track center, reaching the warning value. By applying the mapping relationship between the wheelset coaxial wheel diameter difference and lateral movement from Section 4.1, the wheelset coaxial wheel diameter difference of the vehicle can be reversed to appear in the first wheelset, and the wheelset coaxial wheel diameter difference is about 1.45 mm. By tracking the corresponding vehicle, the wheelset corresponding to the test vehicle was found in the maintenance station, as shown in Figure 37. Through the tread measurement and wheel diameter measurement of the wheelset, it was found that the wheelset coaxial wheel diameter difference appeared, the radius difference was 0.71 mm, and the corresponding diameter difference was 1.42 mm, which was similar to the result derived from the mapping relationship of the system. The error was 2.07%, which verified the reliability of the simulation results in Section 5 and the detection method of the wheelset coaxial wheel diameter difference. In addition, the first and second wheelsets ran to the right, whereas the third and fourth wheelsets ran to the left, and the deviation value was about 1 mm, which did not reach the warning value. It can be seen that differences in the wheel diameters has little influence on different bogies of the same vehicle, which agrees with the simulation analysis results in the previous paper.

**Figure 38:**
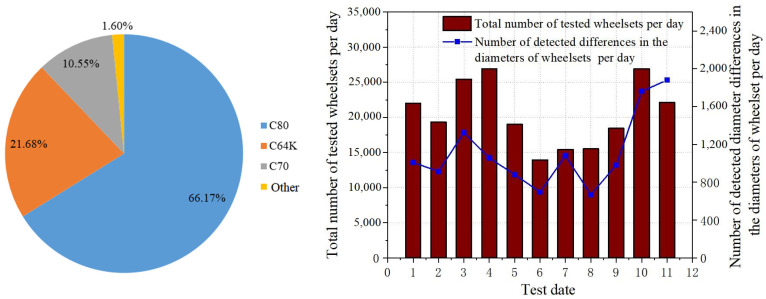
Proportion of vehicles running on the railway (**left**) and the number of wheelsets tested per day (**right**).

Figure 38 shows the proportion of each type of vehicle and the number of wheelsets tested daily. As this railway is a heavy-duty coal line, open wagons are the main means of transport, among which C80 account for 66.17% of the total vehicles, whereas C64K and C70 vehicles account for 21.68% and 10.55%, respectively. The right side of Figure 38 shows the number of wheelsets detected during the test period in 11 days. It can be seen that the average number of past wheelsets detected by the system exceeded 20,000 per day, and the maximum daily detection amount reached 27,000. The proportion of wheelsets detected in a single day with a wheel diameter difference was about 5%, and the proportion detected on the 11th day was a little higher, with about 8.49%. It can be seen that the special railway line was very busy, and the system achieved a better expected effect on the monitoring of passing vehicles.

## 5. Conclusions

Through simulation, the mapping relationship between the wheelset diameter difference and the lateral displacement of wheelset was obtained so as to realize the effect of monitoring the wheelset coaxial wheel diameter difference by using a trackside monitoring system, which can achieve the early warning of wheelset coaxial diameter differences and provide a more intelligent application method for the maintenance of wheelset coaxial diameter differences. First, the characterization and online monitoring methods of the wheelset coaxial wheel diameter difference are analyzed, a reliability analysis of a simulation model is carried out by using the results of simulation calculations and field measurements, and then the mapping relationship between the wheelset coaxial wheel diameter difference and the wheelset lateral displacement is obtained through the simulation. The influence of the vehicle’s offset load on the wheelset coaxial wheel diameter difference detection is eliminated by the analysis. Finally, the field measurement data are applied. The reliability of the mapping relationship and detection method are tested, and the following conclusions can be drawn:

(1) Using the sensors of the simulation software the trackside detection device can be well simulated, and the detection data, which are basically consistent with the trackside detection system, can be obtained. The function of wayside monitoring in the simulation software is realized, and it can better fit the actual situation on-site and achieve better simulation results.
(2) There is an obvious mapping relationship between the wheelset coaxial wheel diameter difference and the wheelset lateral displacement, and the change in the wheelset lateral displacement caused by the wheelset coaxial wheel diameter difference does not change with the vehicle speed. Using the designed wheelset lateral movement trackside detection system and mapping relationship, the wheelset coaxial diameter difference of the tested wheelset can be deduced backwards, the on-time monitoring effect of the wheelset coaxial diameter difference of passing vehicles can be realized, and the fault wheelset can be predicted and warned.
(3) The influence of vehicle off-loading on the wheelset’s lateral displacement is smaller than that of the wheelset coaxial wheel diameter difference, so the influence of vehicle off-loading on the wheelset’s lateral displacement can be basically ignored when identifying the wheelset coaxial wheel diameter difference.
(4) By combining the monitoring results of the trackside monitoring system, a case from all the measured results is selected, the results of the trackside detection system are compared with the faulty wheel failure, and the accuracy of the detection method and mapping relationship is verified.

## Figures and Tables

**Table 1:** Parameters of eddy current sensor.

Items	Parameters
Size	100 mm × 46 mm
Preloader type number	CDS-55VM10
Probe type	ML33-55-00-05
Measuring range	55 mm
Size of the measured object	≥180 × 180 (mm)
Power supply	24 ± 0.01 VDC
Linearity range	8.0 mm–63.0 mm
Independent linearity	1.71%
Midpoint output value	5.206

**Table 2:** Main values of parameters involved in the dynamic model.

Parameters	Units	Values
Car body mass	kg	10,297
Bogie mass	kg	5485
Wheelset mass	kg	1171
Wheelset diameter	m	0.84
Bogie distance	m	8.2
Wheelset base	m	1.83
Wedge spring stiffness	MN/m	0.275
Axial stiffness of intertie	MN/m	14.8
Stiffness of primary suspension along X axis	MN/m	13
Stiffness of primary suspension along Y axis	MN/m	11
Stiffness of primary suspension along Z axis	MN/m	160
Stiffness of secondary suspension along X axis	MN/m	3.127
Stiffness of secondary suspension along Y axis	MN/m	3.127
Stiffness of secondary suspension along Z axis	MN/m	4.235
Car body inertia about X axis	kg·m^2^	1.451 × 10^4^
Car body inertia about Y axis	kg·m^2^	1.06 × 10^5^
Car body inertia about Z axis	kg·m^2^	1.07 × 10^5^

**Table 3:** Summary of mapping relationship between wheelset coaxial wheel diameter difference and wheelset lateral movement.

Wheelset number	First wheelset coaxial wheel diameter difference			Second wheelset coaxial wheel diameter difference		
	a_1_	b_1_	c_1_	a_2_	b_2_	c_2_
1st	−0.1629	1.83054	1.1834	0.12307	−0.99773	0.4178
2nd	−0.0925	0.9669	0.2544	−0.10214	1.46006	−0.0276
3rd	1.68 × 10^−7^	6.78 × 10^−7^	−1.146 × 10^−6^	−3.245 × 10^−7^	2.54 × 10^−6^	−2.372 × 10^−6^
4th	4.878 × 10^−8^	1.11 × 10^−6^	−1.519 × 10^−6^	−3.113 × 10^−7^	2.831 × 10^−6^	−2.272 × 10^−6^
Wheelset number	Third wheelset coaxial wheel diameter difference			Fourth wheelset coaxial wheel diameter difference		
	a_1_	b_1_	c_1_	a_2_	b_2_	c_2_
1st	−1.61 × 10^−7^	1.99 × 10^−6^	−2.863	−3.38 × 10^−7^	3.306 × 10^−6^	−2.64 × 10^−6^
2nd	−1.504 × 10^−7^	1.903 × 10^−6^	−2.732	−3.26 × 10^−7^	2.95 × 10^−6^	−2.542 × 10^−6^
3rd	−0.167	1.8569	1.1032	0.0648	−0.6449	0.1801
4th	−0.107	1.0858	0.114	−0.08857	1.3906	0.01

## Data Availability

Restrictions apply to the availability of these data. Data was obtained from CHINA ENERGY INVESTMENT and are available from the authors with the permission of CHINA ENERGY INVESTMENT.
